# The integrative future of taxonomy

**DOI:** 10.1186/1742-9994-7-16

**Published:** 2010-05-25

**Authors:** José M Padial, Aurélien Miralles, Ignacio De la Riva, Miguel Vences

**Affiliations:** 1 Department of Evolution Genomics and Systematics, Evolutionary Biology Centre (EBC), Uppsala University, Norbyvägen 18D, Uppsala 75236, Sweden; 2 Department of Evolutionary Biology, Zoological Institute, Technical University of Braunschweig, Spielmannstrasse 8, 38106 Braunschweig, Germany; 3 Department of Biodiversity and Evolutionary Biology, Museo Nacional de Ciencias Naturales, CSIC, C/José Gutiérrez Abascal 2, Madrid 28006, Spain

## Abstract

**Background:**

Taxonomy is the biological discipline that identifies, describes, classifies and names extant and extinct species and other taxa. Nowadays, species taxonomy is confronted with the challenge to fully incorporate new theory, methods and data from disciplines that study the origin, limits and evolution of species.

**Results:**

Integrative taxonomy has been proposed as a framework to bring together these conceptual and methodological developments. Here we review perspectives for an integrative taxonomy that directly bear on what species are, how they can be discovered, and how much diversity is on Earth.

**Conclusions:**

We conclude that taxonomy needs to be pluralistic to improve species discovery and description, and to develop novel protocols to produce the much-needed inventory of life in a reasonable time. To cope with the large number of candidate species revealed by molecular studies of eukaryotes, we propose a classification scheme for those units that will facilitate the subsequent assembly of data sets for the formal description of new species under the Linnaean system, and will ultimately integrate the activities of taxonomists and molecular biologists.

## Review

### Taxonomic renaissance

There is little doubt that the central unit for taxonomy is the species, and that associating scientific names unequivocally to species is pivotal for a reliable reference system of biological information [1]. Since the advent of Linnaean nomenclature in 1758, taxonomists have been describing and naming thousands of species every year--currently around 15.000-20.000 among animals only [2,3]--numbers that rapidly increase for many groups of organisms due to the incorporation of new tools for discovery and the exploration of poorly known areas of the planet [4-8]. Indeed, this progress is being made possible despite important impediments [9] because species taxonomy is resurging as a solid scientific discipline [10] that incorporates technological advances, such as virtual access to museum collections [11], high-throughput DNA sequencing [12], computer tomography [13], geographical information systems [14], and multiple functions of the internet [15]. Also, taxonomic information is increasingly digitized and made available through several global initiatives, such as Species2000, The Encyclopaedia of Life (EOL), The Global Biodiversity Information Facility (GBIF), or ZooBank. The future has been envisioned to be an interactive "cybertaxonomy" with dynamic online description and publication of new species, and where updated taxonomic information would be accessible for almost everybody from everywhere [16,17].

However, modern taxonomy still faces two major challenges. First, a qualitative challenge is to reach scientific consensus about the basic category around which taxonomy is built --the species-- and thus improve species delimitation. The second, a quantitative challenge, is the sheer number of species on earth that require discovery and description, estimated in at least 10 million, only considering eukaryotes, and of which a small fraction of less than 2 million have so far been named [18]. These challenges are closely tied to two deliverables that the scientific community and society expects from taxonomy. On one hand, to provide empirical rigor to species hypotheses and stability to their names, which requires a careful and often painstaking and time-consuming labor of species delimitation. On the other hand, an acceleration in the pace of species description, with the peril of erroneous species hypotheses and thus of unstable names.

We here review recent proposals for the development of taxonomy that have been launched to meet both challenges. We argue that recent conceptual advances will allow taxonomy to improve species delimitation through the integration of theory and methods from disciplines studying the origin and evolution of species. Also, we emphasize the importance of applying existing integrative protocols and of developing novel ones for proceeding toward a complete inventory of life on Earth in a reasonable time.

### Integrative taxonomy

Most evolutionary biologists will now agree that species are separately evolving lineages of populations or metapopulations [sensu [19]], with disagreements remaining only about where along the divergence continuum separate lineages should be recognized as distinct species [20,21]. This emerging consensus might appear as a minor advance, but it has led to a renewed discussion about species delimitation that is paramount for catalyzing the integration of new knowledge and methods of population biology, phylogenetics, and other evolutionary disciplines into taxonomy [22-32]. Taxonomists are realizing that what matters for the study of speciation matters for taxonomy as well, and that species will be better delimited if we know what caused their origin and determined their evolutionary trajectories. As illustrative examples, the discovery and description of three Californian species of trapdoor spiders [33] required inferences about the origin, genetic structure and degree of ecological interchangeability of divergent lineages; and a recent taxonomic revision of cardinal birds [34] involved the reconstruction of the populational, phylogenetic and biogeographic history of lineages.

**Figure 1:**
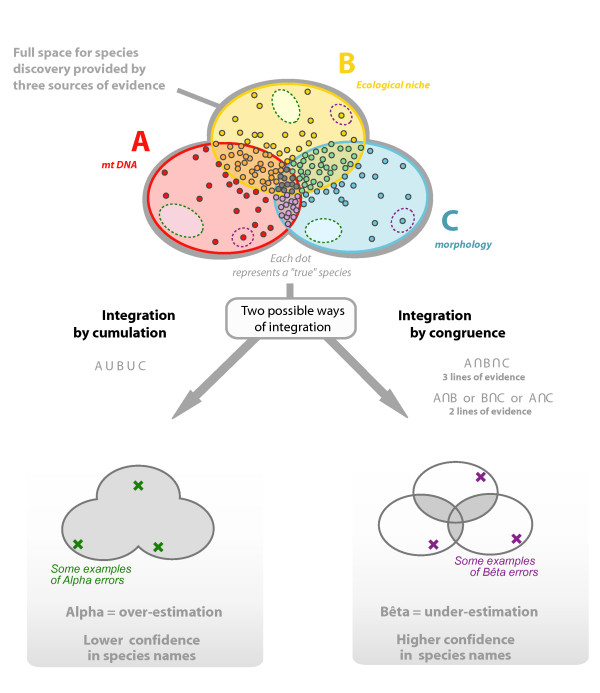
**Schematic representation of two approaches of integrative taxonomy**. Background yellow, red, and blue colors represent the spectrum of character variation, each dot being an independent evolutionary linage that requires identification and delimitation as separate species. Integration by cumulation (left) identifies species limits with divergence in one or more not necessarily overlapping taxonomic characters (e. g. mtDNA *or *morphology), whereas the integration by congruence (right) identifies species limits with the intersection of evidence from two or more independent taxonomic characters (e. g. mtDNA *plus *morphology). Both methods of integration have relevant limitations. The integration by cumulation approach may over-estimate the number of species by identifying distinct species where there is intraspecific character variation only. As an example, conspecific populations can be very distinct in terms of morphology but will be erroneously regarded as distinct species (alpha error or false positive). On the contrary, integration by congruence is a highly stringent approach that might under-estimate the number of species by being unable to detect cryptic or young species (beta error or false negative, represented here by three encircled species among all the undetected ones). Actually, there is a trade-off between the lack of reliability of the species detected by integration by cumulation, and the lower taxonomic resolving power of the integration by congruence.

One consequence of this trend is that after more than 250 years of predominance of comparative morphology in species discovery, new methods and data--mainly molecular--are conquering a great piece of the realm of taxonomy. Although many taxonomists have received with joy the prospects of this new taxonomy [11,12,16,35,36], others are more skeptical [37,38]. Nonetheless, a general view has emerged that now is the time to construct a more "integrative taxonomy" that would accommodate new concepts and methods [36,37,39-41], and a considerable number of studies have already echoed the new term "integrative taxonomy" [28,31,42-45]. A close look at this literature reveals, however, a lack of consensus about what an integrative taxonomy should be. For example, Dayrat [36] proposes to broadly integrate molecular methods and approaches of population genetics at the expense of classical taxonomic procedures, while others hold just the opposite idea [37,38]. Also, major disagreements concern the degree of congruence that different characters must show to consider a population or a group of populations as a separate species. For example, some taxonomists see congruence among molecular and morphological characters as a necessary requisite [36,40,43] while, for others [23,41,46], the strength of integration lies in avoiding any *a priori *selection of character combinations. We here dub these two frameworks as "integration by congruence" and "integration by cumulation", respectively (Figure 1) and explain their advantages and limitations.

### Integration by congruence

**Figure 2:**
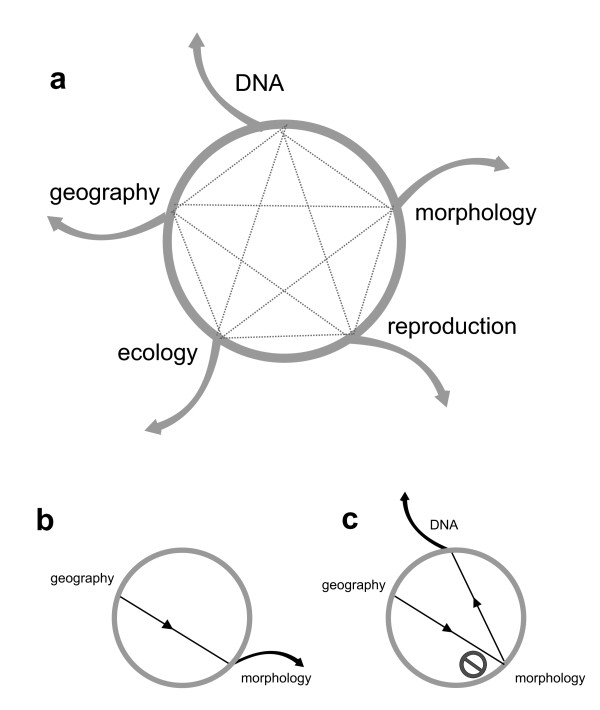
**The "taxonomic circle" representation of work protocol for species recognition**. This protocol (redrawn from [49]) is a schematic representation of the congruence approach to taxonomy proposed by DeSalle and collaborators [49]. Dotted lines in (a) connect lines of evidence used to discover species or support previous hypotheses. The recognition of a species is considered when congruence between a taxonomic character and geography allows breaking out of the circle (arrows). For example, in classical taxonomy (b) the occurrence of morphologically distinct specimens at different locations can be used to propose and support a species hypothesis. In the case of cryptic species (c), morphology fails to support the hypothesis but other characters (e.g. molecular) do provide support.

**Figure 3:**
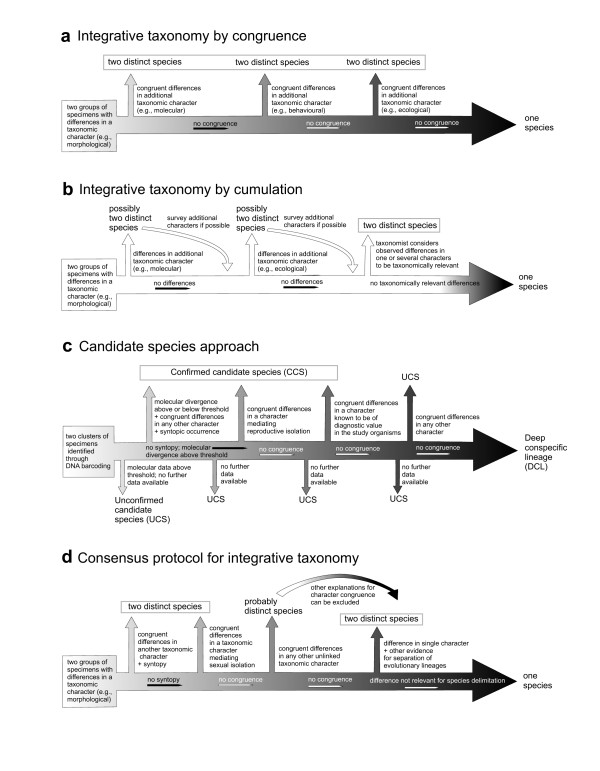
**Schematic representation of work protocols in taxonomy**. Workflow in (a) integrative taxonomy by congruence and (b) by cumulation; (c) work protocol to define Unconfirmed Candidate Species (UCS), Confirmed Candidate Species (CCS) and Deep Conspecific Lineages (DCL) in an automated approach that starts with DNA barcoding [8]; and (d) a general work protocol for integrative taxonomy proposed here that combines advantages of cumulative and congruence approaches. Increasing black color intensity in a-d represents increasing uncertainty about species status and the need of a more thorough evaluation of data.

Congruence approaches have a long tradition in systematics. In phylogenetics, hypotheses of character homology can be examined following the principle of reciprocal illumination, in which each individual character hypothesis is evaluated by the extent to which it agrees with the overall favored phylogenetic hypothesis [47]. In contrast, the congruence approach for species discovery does not examine character hypotheses but lineage divergence hypotheses and can thus most convincingly be founded on population genetics theory [but see [37,38]. In this framework, phylogeographers introduced the genealogic concordance method of phylogenetic species recognition, GCPSR [48]. The GCPSR states that congruent identification of a population-level phylogenetic lineage by several unlinked genetic loci indicates that it is genetically isolated from other such lineages, and thus qualifies as a species, because only in such isolated lineages will the coalescent histories of the different markers agree. Integration by congruence follows the same rationale under the assumption that concordant patterns of divergence among several taxonomic characters indicate full lineage separation. Taxonomists also expect that species so discovered will more often correspond with distinct evolutionary units because it is highly improbable that a coherent pattern of character concordance will emerge by chance. As an example, DeSalle and collaborators [49] illustrated in a work diagram that congruence between two taxonomic characters is an important factor to reach a conclusion about species status (Figure 2a-2c). Different combinations of taxonomic characters may be deemed necessary by different investigators to propose and support species (Figure 3a), such as the congruence of molecular and morphological characters [36,43], or even more restrictive combinations requiring evidence about reproductive isolation [50,51].

**Figure 4:**
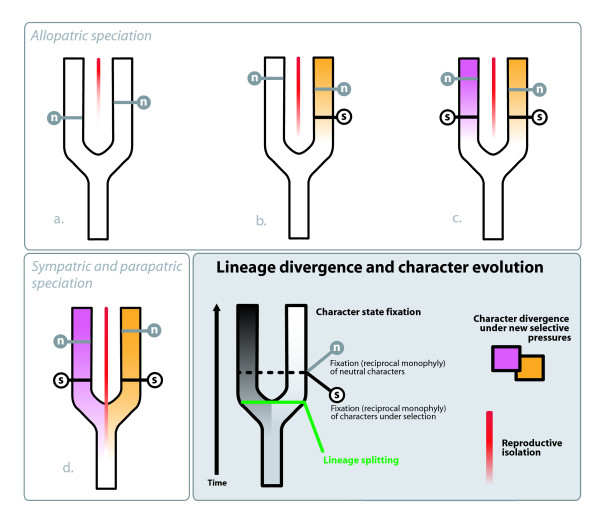
**Schematic representation of character divergence and fixation in several speciation scenarios**. Character divergence varies quantitatively during lineage evolution, with later stages being characterized by marked differentiation (character fixation) at multiple levels, reciprocal monophyly of most gene genealogies, and reproductive isolation [21]. In situations of neutrality or when species are not subjected to novel selective regimes (a, and left lineage of b)-- character fixation is stochastic, due to neutral processes as random genetic drift or because characters are not acquired and fixed in the same order under balancing selection-- a situation known as mutation-order speciation [94]. In cases of speciation along new selective pressures (right lineage of b, c and d), characters subjected to selection are expected to become fixed at early stages of divergence [57,58]. This is especially evident in sympatry or parapatry (c) when disruptive selection causes fitness-dependent character fixation [57,95]. In most scenarios the origin of reproductive incompatibilities will be the result of the epistatic effect of new mutations, under divergent or balancing selection [96]. Reproductive isolation is expected to proceed more rapidly if there is divergent selection, both in allopatry (b, c; [e.g. [97]]), and in sympatry or parapatry (d; [reviewed by [57]]), or in those situations where a character mediating species recognition becomes fixed [77,78]. However, recent evidence indicates that reproductive incompatibilities do not necessarily need to have an adaptive origin, and that they can be the cause of lineage splitting in a neutral or nearly neutral scenario. For example, when the accumulation of new mutations, gene movement, or gene duplication lead to hybrid dysfunction [55]. A textbook-example could be the recurrent cases of polyploidy speciation in plants [98].

The major advantage of the congruence approach is that it promotes taxonomic stability: most taxonomists will agree on the validity of a species supported by several character sets, as long as it is clear that they are unlinked and fixed (see below). The major limitation inherent in demanding congruence among taxonomic characters is the risk of underestimating species numbers (Figure 1) because the process of speciation is not always accompanied by character change at all levels [e.g. [52], and the relative rates of character change during lineage divergence are heterogeneous (Figure 4). Indeed, several empirical studies support the view that lack of character congruence is a frequent situation [reviewed for arthropods by [41]] resulting from the different modes and circumstances of speciation [e.g. [24,53-55]. A further risk of applying integration by congruence could be the bias toward uncovering older species. Species that diverged in the distant past will have an increased probability of showing complete gene lineage sorting and reciprocal monophyly for many loci [56]. The probability of new mutations--and thus character differences--arising and being fixed through adaptive or neutral processes also increases (Figure 4). Furthermore, extinction of relatives creates larger gaps among old species, whereas recent radiations may often be overlooked by a strict consensus approach [53]. For example, in Darwin's finches or some lineages of cichlid fishes, groups of closely related species show striking morphological differences that originated through fast divergent selection associated with ecological transitions, but show weak reproductive isolation, low genotypic clustering, and little neutral genetic differentiation [reviewed by [55]].

### Integration by cumulation

The framework of integration by cumulation is based on the assumption that divergences in any of the organismal attributes that constitute taxonomic characters can provide evidence for the existence of a species [23]. This approach defends the view that because all taxonomic characters are contingent in existence, order of appearance, and magnitude of divergence during speciation, the only way for true integration is allowing any source of evidence--even a single one--to form the basis for species discovery. In this approach congruence is desired but not considered necessary [23]. In practice, evidence from all character sets is assembled cumulatively, concordances and discordances are explained from the evolutionary perspective of the populations under study, and a decision is made based on the available information, which can lead to recognition of a species on the basis of a single set of characters if these characters are considered good indicators of lineage divergence (Figure 3b[41,46]).

A major advantage of this approach is that it does not bind species delimitation to the identification of any particular biological property. Taxonomists can thus select and focus on the most appropriate set of taxonomic characters for each group of organisms. Indeed, this has been the traditional approach of morphological taxonomy (Figure 3b) before the massive incorporation of other characters. Also, cumulation is probably most suitable to uncover recently diverged species in adaptive radiations [53] due to the stepwise process of speciation along ecological gradients [57,58]. The main limitation of the cumulative approach is that the uncritical use of a single line of evidence (e.g. a single locus of mtDNA) can lead to overestimation of species numbers (Figure 1). For example, because of genetic drift, small populations isolated only for a very short period could already become reciprocally monophyletic with respect to some character and be thus diagnosable. Such situations do not represent the type of diversity of interest to most ecologists and evolutionary biologists [59] and the question remains if these populations should be recognized and named as species or not. As an example, Meiri and Mace [50] disagreed with the recognition of Bornean and Sumatran populations of the clouded leopard *Neofelis nebulosa diardi *as a full species [60] for exactly that reason. Indeed, other cases of elevation of subspecies to species rank in several groups of organisms [review by [4]], but especially in birds and primates [61], have been criticized as an unjustified inflation of biodiversity with detrimental consequences for macroecology and conservation [61]. However, in many situations the steep increase of species numbers reflects a genuine discovery of previously unknown evolutionary lineages and thus valuable taxonomic progress [5-7].

### Taxonomic characters

Further discussion about the alternatives for integration requires a closer look to what these approaches try to integrate--the characters. Taxonomic characters are organismal traits used as evidence for species discovery [62]. Characters can (i) be classified by the level of biological organization of the attributes to which they refer: biochemical, molecular, morphological, behavioral or ecological. They can (ii) be qualitative or quantitative, to describe variation that is discrete or continuous. Characters can (iii) have fixed states (that is, for each of the compared species or population there is a unique state for all individuals); or be polymorphic within species but with states distributed in different frequencies across species. Most important for taxonomy--and often-neglected--is the classification of characters (iv) by the evolutionary processes that shaped them (sexual or natural selection, or genetic drift), and by the role they play in the speciation process (Figure 4).

Taxonomists need to resort to different taxonomic characters to conform to the biological peculiarities of particular taxa. For example, behavioral characters--especially those genetically fixed without ontogenetic learning--such as call patterns of insects, bats and frogs, are routinely used by taxonomists working on those groups [e.g. [63]], and their use has led to the discovery of many cryptic species in some groups of organisms [64]. Also, the ecology of organisms can be an important source of evidence in some cases. For example, the degree of ecological interchangeability may be a decisive taxonomic character to distinguish between closely related species [26,33,65]. In bacteria, the lack of a conspicuous morphology coupled with extensive gene transfer has forced taxonomists to develop a model-based strategy that combines data on ecology and on genetic diversity to delimit species [66,67].

However, much of the discussion around integrative taxonomy deals with the merits of morphological versus molecular characters [36-39,41,49]. For practical and historical reasons most species have been primarily described based on morphology, including color. As a main advantage, morphological characters often serve to allocate individuals to species immediately by visual inspection, and are applicable to living as well as preserved specimens and fossils. Disadvantages are: (i) there is always a subjective component when defining and interpreting character states, (ii) demonstrating the fixation of a state requires large sample sizes [68], (iii) the continuous rather than discrete nature of many characters on which taxonomists heavily rely, e.g., reptile scale counts [69] or mollusk shell size and shape [70], and (iv) their unsuitability for some groups of organisms, either because speciation occurs without morphological change [52], which leads to morphologically cryptic species [64], or because morphological structures are labile or difficult to study--e.g. as in prokaryotes [66,67].

Molecular characters used in taxonomy have historically been allozymic or chromosomal (number and structure of chromosomes), but today these are mostly sequences of mitochondrial (or chloroplast) DNA and, increasingly, of nuclear genes. While the analysis of allozymes has the advantage of simultaneously screening variation at several presumably unlinked nuclear loci, DNA sequences provide many more characters (nucleotide sites), can be amplified from much smaller samples, and can be obtained in unprecedented amounts through high-throughput sequencing from fresh samples, preserved historical collection material, and even from Pleistocene fossils [71]. DNA sequences can be examined using non-tree based methods to provide diagnostic differences among species [49], but most frequently they are analyzed using tree-based methods to search for monophyletic groups that could represent species [22]. A limitation of tree-based methods is that it remains difficult to choose which among the multiple strongly supported clades detected represent species. Also, a growing body of evidence shows that discordance between species trees and gene trees is a common phenomenon caused by processes such as incomplete lineage sorting, hybridization, gene duplication, reticulated evolution, or recombination [54]. These situations greatly complicate the resolution of taxonomic problems [72,73]. Most promising are tree-based methods that rely on coalescent theory [54,74], because they can identify signals of species divergence even under complex circumstances of gene tree incongruence and non-monophyly [e.g. [26]]. A recent large-scale study on insects of Madagascar [75] shows that single-locus coalescent models perform well for both testing and discovering species from large sample sets even without prior hypotheses of population coherence, providing thus a potential empirical substitute for traditional tree-based methods for preliminary biodiversity screening and species identification (e.g., DNA barcoding approaches).

### The merits of taxonomic characters under the integrative taxonomy framework

To be usable in an integrative taxonomy, characters should be evaluated taking into account the evolutionary forces driving the speciation process (Figure 4). This new perspective may help to overcome many of the long-standing discussions about characters in taxonomy. For example, as long as characters have a genetic basis, are unlinked and are not influenced by the same selective pressures, any character should be considered as an independent, equivalent and combinable unit. In other words, the potential of a character to clarify a taxonomic problem has to be carefully evaluated in every situation, and a particular nucleotide or morphological character state might be deemed more important than all other nucleotides and morphological characters, because it might be particularly informative to understand the process of lineage splitting and divergence. For instance, in those cases where the speciation process is driven by sexual and/or by natural selection, characters known to be under the influence of any of these forces in one species might be directly indicative of lineage divergence in the whole taxonomic group to which this species belongs and, thus, be more informative than those that are known to evolve neutrally.

Such inferences need to be based on careful evaluations to avoid circularity of arguments. As a very obvious example, in a group of animals where speciation has been demonstrated to be mainly driven by sexual selection of male colouration, differences in colour will be given a higher importance as taxonomic character than in a group of subterraneous and blind species. If speciation in a clade of phytophagous insects is known to be driven mainly by the switch to new host plants, then the discovery that a new population belonging to this clade feeds on a novel host plant might be a more relevant taxonomic character than it would be in a clade of host plant generalists. And in a group of microendemic species where specialization to narrow and distinct bioclimatic envelopes has been demonstrated to be the main force leading to speciation, the specialization of a newly discovered population to a bioclimatic niche distinct from all known species in the group might be a suitable argument to advocate its species status, while in groups where most species are known to be tolerant of a variety of bioclimates such data would be less relevant.

In general, sexually selected characters might be more likely to represent species-specific differences than naturally selected characters because they contribute to the reproductive cohesion and isolation of species while, at the same time, their rapid evolution contribute to create larger gaps between closely related species. Thus, the most important taxonomic characters would be those indicative of reproductive isolation or limited gene flow, such as crucial mutations in "speciation genes" [76] or any trait that directly mediates a premating or postmating reproductive barrier, such as wave form and frequency differences in advertisement calls of insects or frogs, or genital structures of arthropods and squamate reptiles. For example, a single amino acid substitution can suffice to produce striking plumage differences mediating species recognition and leading to speciation in birds [77]. Also, differences in a coding vision gene can affect female mating preferences and initiate lineage divergence in fish [78]. Some researchers further argue that molecular markers prone to high intraspecific gene flow might be less affected by interspecific gene flow, and be thus more effective for delimiting species [27], because in cases of gene introgression among sister species substantial intraspecific gene flow will reduce the frequency of introgressed alleles. But, also, the first move in speciation can result of the accumulation of multiple neutral mutations in DNA sequences causing hybrid incompatibilities [55], which make those mutations ideal diagnostic characters to separate species.

In short, future discussions in taxonomy should not be about morphology versus molecules but about how characters reflect lineage divergence or about the functional relevance of some characters in the speciation process. As a consequence, taxonomy will no longer be a science restricted to the description of patterns but will be tightly linked to the study of processes generating diversity.

### Candidate species

In the practice of current (increasingly molecular) systematics, phylogeography and DNA barcoding studies of eukaryotes are revealing units that might represent potential new species at a faster pace than results can be followed up by taxonomists. This situation suggests a need for guidelines to order and classify this undescribed diversity. The bacteriological concept of candidate species [79] has recently been explored and applied to vertebrates for such units [8,63,80,81]. A further developed stepwise working protocol (Figure 2c) recognizes three subcategories of candidate species [8]. Groups of individuals within nominal species showing large genetic distances, but without further information, are considered unconfirmed candidate species (UCS) deserving further study. When additional data indicate that these genealogical units are not differentiated at the species level, they are flagged as deep conspecific lineages (DCL). The third category, confirmed candidate species (CCS), applies to those deep genealogical lineages that can be considered good species following standards of divergence for the group under study but that have not yet been formally described and named. For example, confirmed candidate species are sister lineages in syntopy showing no evidence of interbreeding, or allopatric lineages with distinct morphological or bioacoustical character divergences.

A more standardized nomenclatural system might help to communicate with precision about candidate species, inventory them and track their changing status. Murray and Schleifer [79] proposed a formal naming system for candidate prokaryotes that consists in placing the epithet *Candidatus *before a preliminary species name, as for example: "*Candidatus *Liberobater asiaticus". This system has since been broadly accepted and implemented in the Bacteriological Code. However, it is inapplicable for eukaryotes because the zoological and the botanical codes do not specify minimum scientific criteria for recognizing species names as valid. Thus, while "*Candidatus *Liberobater asiaticus" can be kept as an informal name until the species is proved to be valid by accepted standards of the discipline and becomes thus formally described, any informal name given to a candidate species of animal could qualify as valid name under the Zoological Code if the proposal is accompanied by a voucher and diagnostic differences (e.g. exclusive haplotypes).

For different groups of animals, naming schemes of candidate species have been established. As one example, catfishes of the family Loricariidae are provided with so-called L-numbers, a system of consecutive numbers introduced in 1988 by R. Stawikowski, A. Werner and U. Schliewen, in which each putative new species is referred by a unique number combination after the letter "L"--from Loricariids. These numbers are designated upon publication of photographs of unknown color variants of Loricariids in the German journal "Die Aquarien und Terrarien Zeitschrift" and are used beyond the realm of aquariologists.

A standardization of such schemes across taxonomic groups of eukaryotes would be clear progress for data retrieval systems. A naming scheme for candidate species should not be mistaken for a substitute or competition with the established Linnaean system of nomenclature but, rather, it is a mean to facilitate the assembly of data sets that could eventually lead to the description of new species under the Linnaean system. To avoid conflicts with the Codes of nomenclature, we propose to designate candidate species of eukaryotes through the combination of the binomial species name of the most similar or closely related nominal species, followed (in square brackets) by the abbreviation "Ca" (for candidate) with an attached numerical code referring to the particular candidate species (more than one candidate might be recognized under a valid species), and terminating with the author name and year of publication of the article in which the lineage was first discovered. The vouchers the candidate species cfould be the GenBank accession numbers of the sequences used to propose the candidate status, or any equivalent information (e.g. MorphoBank accession numbers for morphological candidate species, or a voucher specimen number from a public collection). As an example, *Hirudo medicinalis *[Ca3 Siddall et al. 2007], would be the exclusive name combination referring to a particular candidate species of European leech (Ca3), as defined in the corresponding reference [82]. This system should be flexible enough to accommodate situations in which no unambiguous candidate species definition has been proposed in a study--in this case, referring to a GenBank accession number should be possible: *Hirudo medicinalis *[Ca3 EF405599], where the number refers to a highly divergent sequence of *H. medicinalis*. When not even a tentative assignment of a candidate species to a most similar nominal species is possible, then it would be possible to assign a candidate species just to a genus or family, i.e., *Hirudo *sp. [Ca3 EF405599] or Hirudinidae sp. [Ca3 EF405599].

This system maintains the traditional structure of binomial species names and helps to list together both valid and candidate species in hierarchical and alphabetically ordered databases as GenBank, or repositories of morphological or geographical data. Adding a numerical code helps to avoid repetitive proposal of candidates; and GenBank accession numbers provide a direct link to source data. Candidate species should create a link between the activities of molecular biologists (e.g. ongoing DNA barcoding initiatives) and taxonomists to redirect taxonomic efforts and accelerate species descriptions.

## Conclusions and future perspectives for an integrative taxonomy

The past years have seen an important reduction of taxonomic impediments: conflicts about species concepts are being replaced by a consensus on the view of species as lineages [see [25]]; the access to crucial taxonomic information is facilitated by a growing body of cyber-infrastructures such as species names databases, digitized original descriptions, and online imagery of type specimens and historical literature [83]; the enormously successful journal Zootaxa provides an example of a fast platform for the publication of zoological taxonomic studies [3]; widespread use of DNA sequence data has led taxonomy to an enormous acceleration in the identification of cryptic species [review by [64]] and candidate species [8,80,81,84-88]. We hypothesize that those advances have overall contributed species description rates to remain roughly stable at 14,000-25,000 per year since 1970 [89] despite a decrease in the number of taxonomists [90]. However, if estimates of 10 million eukaryote species on Earth were correct [18], we would need, at the present pace, some 400 years of constant activity of taxonomic research to attain a "complete" inventory of life. This is not a reasonable time given that human activity is driving species to irremediable extinction at a rate never experienced before [91]. We identify five major working lines for the scientific development of integrative taxonomy that will accelerate species description without compromising accuracy [cf. [92]] (Table 1). However, the success will not exclusively depend on the development of new concepts and approaches. Most importantly, more taxonomists and more funding for taxonomy are urgently needed [90]. A stronger focus on actually publishing systematic revisions and species descriptions should, too, be part of a strategy to alleviate the taxonomic impediment [93].

**Table 1:** Work areas for the scientific and technical development of integrative taxonomy

Improving taxonomic work protocols	Development of pragmatic operational protocols to discovering and describing species (Figure 2d). There is an inevitable trade-off between using complex integrative approaches for delimiting species that may provide stable names, and the need to accelerate the pace of taxonomic descriptions [92]. Indeed, of the many empirical methods available for species delimitation [22,25], most require extensive sampling, absence of missing data, and/or complete species-level molecular phylogenetic trees. Clearly, for most areas and groups of diverse organisms of the world, data at hand will be insufficient for in-depth studies of evolutionary separation of lineages.
	
Refinement of probabilistic procedures to evaluate character congruence	New methods should be able to deal with the heterogeneity of the evolutionary process, with situations of character incongruence, and to include fixed characters states as well as states distributed in different frequencies across populations. In this sense population geneticists have efficient tools to estimate if combinations of alleles occur more frequently than expected randomly -a situation termed linkage disequilibrium [99]- and this method can be applied to discover cryptic sympatric species [e.g., [100]]. Also, phylogeneticists have developed approaches as CONCATERPILLAR [101], which take into account different evolutionary rates of different loci and allow identification of those that should be analyzed independently or concatenated. Extending such approaches to non-molecular characters could result in more rigorous protocols of integrative taxonomy.
	
Development of modular software for species delimitation, description and publishing.	Besides including phylogenetic and population genetics modules, as in Mesquite [102], such software should include modules for statistical analysis of morphological data, should be able to extract character information from bi- and tri-dimensional imagery [103] and from sequence data (such as pure and private diagnostic nucleotide substitutions, e.g. [49]), and should also incorporate packages for ecological and geographical modeling and mapping, as well as for bioacoustics. It could also implement a package for building standardized species descriptions that could be directly submitted for peer-review to major taxonomic journals at the same time that supporting data are automatically sent to biodiversity databases (e.g. GBIF, Species2000, Zoobank, GenBank, CBOL, MorphoBank); hyperlinked species descriptions represent an advance in this sense [13].
	
Automated identification of candidate species	Development of methods for automatically identifying, naming, documenting and cataloguing candidate species through the combination of DNA barcoding and digital image processing [12,103]. These approaches could be especially helpful for the preliminary screening of hyperdiverse groups such as small arthropods and nematodes, or for geographical areas facing imminent habitat destruction (and therefore in need of rapid inventories of species diversity and conservation priorities).
	
Application of genomic analyses to taxonomy (GenoTaxonomy).	Population genomics aims to identify regions of the genome with greater differentiation than expected from the average across many loci affected by reduced gene flow due to reproductive isolation or local adaptation [104]. The automatic identification of those regions, to be used as diagnostic characters, might be the key to substantially accelerate species discovery, especially if applicable through modular taxonomic software. Given the enormous expected increases of genomic data [105] such approaches will soon become applicable.

Although traditional procedures will remain useful in many cases, taxonomy needs to be pluralistic and integrate new approaches for species delimitation if it is to become a modern evolutionary discipline. Thus, for example, the "Family Union"--in Joe Felsenstein's words--between the fields of population genetics and phylogeography with phylogenetics through coalescent theory, which has been considered as one of the most exciting recent developments in systematics [69] (see also http://treethinkers.blogspot.com/), should also strongly benefit taxonomy. Shadows of past conflicts between morphologists and molecular biologists should now fade and discussions will not be about simply integrating different kinds of characters, but rather different concepts and methods of population genetic, phylogeographic, and phylogenetic analyses. There is probably no magic bullet for species discovery and delimitation, but an integrative and evolutionary framework provides taxonomists with a larger arsenal to face the realities of inventorying the actual--and woefully underestimated--biodiversity of the planet.

## Competing interests

The authors declare that they have no competing interests.

## Authors' contributions

JMP and IDLR developed the concept of this review. JMP, AM and MV drafted the manuscript and the figures. All authors intensively revised subsequent drafts of the manuscript and read and approved its final version.

## Appendix

### Glossary of selected terms used in the text

**Allele: **one of two or more alternative forms of a gene that arise by mutation and are found at the same locus in a chromosome.

**Allopatry: **the condition of species or populations occurring in separate, non-overlapping geographical areas.

**Candidate species: **a set of organisms identified as a putative new species.

**Coalescent theory: **retrospective model of population genetics that employs a sample of individuals from a population to trace all alleles to the most recent common ancestor.

**DNA barcoding: **the use of short standardized DNA sequences to identify species.

**Ecological niche: **environmental conditions under which a species exist.

**Gene lineage: **ancestor-descendant series of alleles.

**Locus (plural loci): **a fixed position on a chromosome that may be occupied by one or more alleles of a gene.

**Monophyletic group: **a group consisting of an ancestor and all its descendants.

**Neutral character: **observable or quantifiable organismal trait whose evolution and variation can be explained by random processes.

**Non-neutral character: **observable or quantifiable organismal trait whose evolution and variation can be explained by natural or sexual selection.

**Parapatry: **the condition of species or populations occurring in contiguous geographical areas.

**Paraphyletic group: **a group consisting of an ancestor and some of its descendants.

**Phylogenetics: **biological discipline focused on reconstruction of the evolutionary relationships among organisms.

**Phylogeography: **study of historical processes responsible for intraspecific patterns (or patterns among closely-related species) of geographical distribution and diversity of gene lineages.

**Species hypothesis: **the hypothesis that a group of populations represents a separately evolving and divergent lineage.

**Species lineage: **ancestor-descendant series of metapopulations.

**Speciation: **the array of processes that result in the origination of new species.

**Subspecies: **infraspecific Linnaean category sometimes used to classify allopatric or parapatric populations showing some degree of divergence--traditionally in morphological traits--not considered large enough for the species rank.

**Sympatry: **the condition of species occurring in the same geographical area.

**Syntopy: **the condition of species occurring in the same locality at the same time.

**Systematics: **biological discipline that studies evolutionary patterns of biological diversity, including the fields of taxonomy and phylogenetics.

**Taxonomy: **biological discipline that identifies, describes, classifies and names extant and extinct living beings and deals with the theory of classification.

**Taxonomic character: **observable or quantifiable organismal trait used to separate species.
